# Foreign Body in the Lung; Report of Case1Read at the thirty-eighth annual meeting of the Medical Association of Central New York, held at Buffalo, October 24, 1905.

**Published:** 1906-03

**Authors:** Francis E. Fronczak

**Affiliations:** Buffalo. N. Y.


### Foreign Body in the Lung; Report of Case.[1]

By FRANCIS E. FRONCZAK, A.M., M. D., Buffalo. N. Y.

ON the sixth day of July, 1905, I was called to see a child about 8 years old, who was said to be choking. The little patient, a girl, was in bed, somewhat cyanotic and breathing with considerable difficulty, about 32 times per minute, and coughing almost continually. This attack, as described by the mother of the girl, was quite sudden, the child being perfectly well a few hours before. On examination I found the throat to be perfectly clear and absolutely normal in every respect. On percussion of the chest the right lung gave a dull sound; on auscultation there were found many rales on the right side of the chest,—the left having no abnormal signs of any kind. The elevation of temperature was very slight. The child denied swallowing or aspirating anything, though I suspected some foreign body either in the bronchus or the lung. Removal to the hospital was proposed but declined. I saw the child several times, but there was no change in the condition, the physical signs remaining the same as on my first visit. The rales, though, were very much in evidence, so that they could be heard without the sthethoscope.

Two months afterward, that is, on September 12, the girl had a very violent fit of coughing and after some time coughed up a melon seed, somewhat decayed. I learned later that on the day this girl became ill, she was eating a melon and presumably aspirated a seed which at times threatened her life. The fits of cough stopped almost immediately, the rales disappeared within few days, and the child who was losing both color and weight,

---

1. Read at the thirty-eighth annual meeting of the Medical Association of Central New York, held at Buffalo, October 24, 1905.

began to recover lost ground, and at present has the appearance of a perfectly healthy school-girl.

The points which interested me in this case were: the length of time the foreign body lodged in the lung, over two months; the sudden development of the symptoms and their speedy disappearance when the object was dislodged from the lung, where, I believe it was located; and lastly, it taught me a very useful lesson,—namely, not to make a prognosis too grave. I told the mother after the child denied swallowing or aspirating anything, and not improving under treatment, that she had probably developed some disease of the lung, tubercular, and that she would not recover. I was wrong in both of my deductions and diagnoses, of which fact I am glad. It is human to err, but medical men are not often forgiven their errors.

508 FILLMORE AVENUE.

